# Evidence-based biomedical research in Sub-Saharan Africa: how library and information science professionals contribute to systematic reviews and meta-analyses

**DOI:** 10.5195/jmla.2022.1249

**Published:** 2022-01-01

**Authors:** Toluwase Victor Asubiaro, Elueze Isioma

**Affiliations:** 1 tasubiar@uwo.ca, https://www.fims.uwo.ca/people/profiles/toluwase_asubiaro.html, Doctoral Candidate (formerly), Library and Information Science Program, Faculty of Information and Media Studies, University of Western Ontario, London, Ontario, Canada; 2 ielueze@fanshawec.ca, Fanshawe College, London, Ontario, Canada

**Keywords:** Sub-Saharan Africa, systematic reviews, meta-analysis, information professionals, evidence-based biomedical research, content analysis, librarians

## Abstract

**Objective:**

This study investigated the contributions of library and information science (LIS) professionals to systematic reviews and meta-analyses with authors from Sub-Saharan Africa. It also investigated how the first author's address and type of collaboration affected the involvement of LIS professionals in systematic reviews and meta-analyses.

**Methods:**

Bibliographic data of systematic reviews with author(s) from the forty-six Sub-Saharan African countries was retrieved from MEDLINE. Content and bibliometric analyses were performed on the systematic reviews' full-texts and bibliographic data, respectively, to identify the contributions of LIS professionals and collaboration patterns.

**Results:**

Beyond traditional roles as search strategy developers and searchers, the LIS professionals participated in article retrieval, database selection, reference management, draft review, review conceptualization, manuscript writing, technical support, article screening and selection, data extraction, abstract review, and training/teaching. Of the 2,539 publications, LIS professionals were mentioned in 472 publications. LIS professionals from only seven of the forty-six Sub-Saharan African countries were noted to have contributed. LIS professionals from South Africa were mentioned most frequently—five times more than those from other Sub-Saharan African countries. LIS professionals from Sub-Saharan Africa mostly contributed to publications with first authors from Sub-Saharan Africa (90.20%) and intra-Sub-Saharan African collaboration (61.66%). Most LIS professionals (97.91%) that contributed to international collaboration publications were from outside Sub-Saharan Africa.

**Conclusion:**

The contribution of LIS professionals in Sub-Saharan Africa to evidence-based biomedical research can improve through training, mentoring, and collaboration between LIS associations in Sub-Saharan Africa and those in countries with resources and a history of research collaboration with the region.

## INTRODUCTION

Health sciences library and information science (LIS) professionals provide many services, carry out different activities, and play various roles in the context of their work. Some of these activities and roles include liaison, informationist, data management, reference, research, and instruction. Other roles are captured in nomenclatures like teacher, technology specialist, embedded librarian, information consultant, knowledge manager, and subject librarian [1]. In particular, LIS professionals have much to contribute to research, as they have a broad knowledgebase and skill set that can be applied to research in many areas [2]. More specifically, LIS professionals have a role to play in conducting systematic reviews, which attempt to “collate all empirical evidence that fits pre-specified eligibility criteria in order to answer a specific research question” [3], and are an important type of research that promotes evidence-based practice.

McGowan and Sampson found that expert searchers are an essential part of the systematic review team and are crucial throughout the review process, from the development of the protocol and review question to publication [2]. Morris, Boruff, and Gore found that LIS professionals contribute to systematic reviews and meta-analyses as expert searchers, methodologists, and information managers; thus, their central roles in systematic reviews go beyond searching [4,5] and also include information management, formulation of review questions, development of search or information retrieval strategies, results collation, and report writing [5]. Furthermore, Harris [6] reported that librarians' multiple roles as expert searchers, organizers, and analyzers form an integral part of the Cochrane Collaboration's criteria for conducting systematic reviews.

Many studies have examined the roles of LIS professionals in the systematic review process [4, 5, 7–10]. However, none of these studies focused on regions with a scarcity of resources, such as Sub-Saharan Africa. This study fills this gap in the literature by analyzing published systematic reviews and meta-analyses with authors from Sub-Saharan African countries to understand the contribution of LIS professionals to evidence-based research in this region. The literature shows that there are peculiarities in the research systems of Sub-Saharan African countries because of the challenges of low funding, resource scarcity, and dearth of on-the-job training. Therefore, research systems in Sub-Saharan Africa are strongly influenced by foreign countries. Hence, we also investigated the influence of LIS professionals in foreign countries on evidence-based research in the region by analyzing the country affiliation of LIS professionals who contributed to systematic reviews and meta-analyses. The specific objectives of the study were:

1. To investigate the contributions of LIS professionals within and outside Sub-Saharan Africa in systematic reviews and meta-analyses with at least one author from the region;
2. To investigate the relationship between contributing LIS professionals' affiliations and the first author's affiliation; and
3. To investigate the relationship between contributing LIS professionals' affiliations and the type of collaboration.

## METHODS

### Data collection

This study was exempt from review by the institutional review board because data were collected from publicly available publications. Bibliographic data for biomedical systematic reviews and meta-analyses published between 2014 and 2019 with at least one author affiliated with an institution in Sub-Saharan Africa were retrieved from MEDLINE via the PubMed search engine. The names of all forty-six countries in Sub-Saharan Africa were included in the search query as affiliation (Appendix A). Publication types were specified as systematic reviews and meta-analyses as delimiters, and text word search was also employed so that systematic reviews and meta-analyses that mentioned their publication type in their abstract or keywords but were not indexed accordingly could be retrieved. The publication year range was set to 2014 to 2019 because MEDLINE started to include affiliation information of all authors in 2014, whereas only first author affiliations were indexed before this time [11].

MEDLINE is an authoritative and specialized database that indexes biomedical publications. MEDLINE has advantages because of search affordances such as MeSH search capabilities that are not available in other authoritative scholarly databases such as Scopus, Web of Science, and Africa Index Medicus [12]. However, as studies report bias of these databases against publications from Africa, this indicates that the dataset for this study may not include all relevant publications from the Sub-Saharan African region [13,14]. African Journals Online is an alternative database, but its advanced search capacity is not well developed, and it indexes only a fraction of journals published in Africa.

Publications were included if they were systematic reviews, systematic review protocols, and/or meta-analyses with at least one author from a Sub-Saharan African country. Commentaries, corrections, articles about systematic review methodology, and meta-analyses that did not include systematic review methodology were excluded.

### Data extraction and content analysis

The following data were recorded in a spreadsheet for each publication: article title, authors, journal title, full-text availability, systematic review/meta-analysis, LIS professionals mentioned, section of article in which LIS professionals were mentioned, quoted text describing LIS professionals' contributions, and LIS professionals' institutional and country affiliations.

Specific sections of the full texts perused were the author list, methodology, acknowledgments, and authors' contribution statement (if available). These sections were read for mentions of terms that were used synonymously with LIS professionals, including information specialist, informationist, information scientist, medical librarian, search specialist, health science librarian, resource center manager, literature search specialist, information services provider, and librarian. Mentions of a library for mundane reasons (e.g., Internet use) were also noted.

Following data extraction, content analysis was performed, whereby a list of roles was generated by thoroughly reading through and coding quoted text from the publications in which an LIS professional was mentioned or that described the contributions of an LIS professional. This process involved open coding that remained close to the text and included memo writing and reflection [15]. As Corbin and Strauss [16] stress the value of employing existing frameworks for informing the data analysis process in qualitative work, we relied on roles attributed to LIS professionals from previous studies [2, 4, 6, 8, 10] as a sensitizing tool. However, the coding process was also open to allow the emergence of new roles. Role classification was not mutually exclusive, as some systematic reviews had LIS professionals' roles classified into more than one category. Similarly, some studies referred to LIS professionals in multiple sections of the full text (e.g., in the methodology, acknowledgments, and author list). Although only systematic reviews and meta-analyses that included authors from Sub-Saharan African countries were included in this study, all LIS professionals' contributions were classified regardless of their country affiliation.

### Bibliometric analysis

Bibliometric data on the first author's country affiliation, LIS professionals' country affiliations, and collaboration type were collected from publications that contained LIS professionals' contribution to investigate the relationships between the first author's country affiliation and collaboration type and the country affiliation of contributing LIS professionals. LIS professionals' and the first author's country affiliations were coded as “Sub-Saharan Africa,” “outside Sub-Saharan Africa,” or “hybrid.” The first author's country affiliation was coded as “Sub-Saharan Africa” if the first author was affiliated with institution(s) within Sub-Saharan Africa and not those outside the region, “outside Sub-Saharan Africa” if the first author was affiliated with institutions outside the region and not those within the region, and “hybrid” if the first author was affiliated with institutions both within and outside Sub-Saharan Africa. Collaboration type was coded as “no collaboration,” “institutional collaboration,” “national collaboration,” “intra-African collaboration,” or “international collaboration.” Collaboration type was coded as “no collaboration” if there was only one author, “institutional collaboration” if all authors were affiliated with the same institution, “national collaboration” if authors were affiliated with more than one institution in one Sub-Saharan African country, “intra-African collaboration” if authors were affiliated with institutions in two or more Sub-Saharan African countries, and “international collaboration” if authors were affiliated with institutions both within and outside Sub-Saharan Africa. Spearman's rho correlation analysis was performed to determine the relationship between the country affiliations of contributing LIS professionals and the first author and the relationship between the country affiliations of contributing LIS professionals and collaboration type.

## RESULTS

Out of 3,171 publications that were retrieved initially, 632 were excluded (full text of 82 publications were unavailable and 550 did not meet inclusion criteria). The remaining 2,539 systematic reviews and meta-analyses (208 of which were systematic review protocols) were included. Of these, LIS professionals were mentioned in 472 publications (417 systematic reviews and meta-analysis and 55 protocols). LIS professionals were mentioned in the acknowledgments in 251 studies, the methodology in 242 studies, the author list in 79 studies, and as a member of an author group in 1 study.

### Number of systematic reviews and meta-analyses with LIS professionals' contributions

Of the 472 systematic reviews and meta-analyses that mentioned the contribution of LIS professionals, addresses of the LIS professionals were not specified in 177 publications. Of the remaining 295 publications, 192 and 104 mentioned LIS professionals from institutions outside and within Sub-Saharan Africa, respectively (Table 1).

**Table 1:** Contribution of LIS professionals from countries within and outside Sub-Saharan Africa

	Countries within Sub-Saharan Africa				Countries outside Sub-Saharan Africa			
S/N	Country	Total number of publications	Number of publications with LIS professionals' contribution	% of publications with LIS professionals' contribution (out of 295)	Country	Total number of publications	Number of publications with LIS professionals' contribution	% of publications with LIS professionals' contribution (out of 295)
1	South Africa	1,118	68	23.05	USA	670	39	13.22
2	Ethiopia	445	12	4.07	UK	664	43	14.58
3	Nigeria	308	8	2.71	Australia	363	30	10.17
4	Kenya	204	2	0.68	Canada	221	26	8.81
5	Cameroon	156	0	0	The Netherlands	213	27	9.15
6	Ghana	148	0	0	Switzerland	196	5	1.69
7	Uganda	145	12	4.07	Germany	153	4	1.36
8	Tanzania	114	1	0.34	France	142	0	0
9	Zimbabwe	47	0	0	Belgium	125	2	0.68
10	Malawi	35	0	0	Italy	101	0	0
11	Zambia	31	0	0	India	93	0	0
12	Mozambique	29	1	0.34	Brazil	87	0	0
13	The Gambia	29	0	0	Sweden	84	1	0.34
14	Rwanda	24	0	0	China	81	3	1.02
15	Burkina Faso	21	0	0	Spain	69	0	0
16	Senegal	16	0	0	Norway	58	4	1.36
17	Dem. Rep. Congo	15	0	0	Iran	53	4	1.36
18	Gabon	15	0	0	Japan	48	1	0.34
19	Benin	14	0	0	Denmark	45	2	0.68
20	Cote D'Ivoire	14	0	0	Malaysia	33	1	0.34
21	Botswana	13	0	0	New Zealand	30	0	0
22	Namibia	10	0	0	Thailand	30	0	0
23	Congo	9	0	0	Singapore	27	0	0
24	Mali	8	0	0	Bangladesh	25	0	0
25	Togo	7	0	0	Portugal	24	0	0

LIS professionals did not contribute to systematic reviews and meta-analyses in some highly productive countries such as Cameroon, Ghana, Zimbabwe, Malawi, and Zambia (Table 1). Rather, LIS professionals contributed to systematic reviews and meta-analyses in only seven Sub-Saharan African countries (South Africa, Ethiopia, Nigeria, Kenya, Uganda, Tanzania, and Mozambique). LIS professionals in Ethiopia, Uganda and South Africa were the most productive among the Sub-Saharan African countries, as they contributed to 31% of the regional output on systematic reviews and meta-analyses. LIS professionals from countries outside Sub-Saharan Africa were most often affiliated with the United Kingdom, the United States, Australia, the Netherlands, and Canada and contributed to 56% of the regional output on systematic reviews and meta-analyses.

### Contributions made by LIS professionals to systematic reviews and meta-analyses

Of the 472 included publications, details of the contributions of the LIS professionals were not provided in 58 publications. The following are three examples of roles classified as “non-specific”:

“Our sincere gratitude also goes to the Mozambique Ministry of Health Library staff for their unrelenting assistance.”

“The authors gratefully acknowledge the College of Health Sciences at the University of KwaZulu-Natal and the Medical Liberian for the support.”

“The authors acknowledge Ms. Dilshaad Brey the UCT Libraries, Health Sciences, and Information Services Librarian”

The specific contributions of LIS professionals to the remaining 414 publications were classified as described below.

#### 1. Developing search strategy (199 publications)

Most LIS professionals, including those from South Africa (n=23), Uganda (n=3), and Nigeria (n=1), helped in the development of search strategies for systematic reviews and meta-analyses. Some other terminologies used for the involvement of LIS professionals in developing a search strategy were testing the search strategy, reviewing the search strategy, assessing the quality of the search strategy, validating the search strategy, identifying keywords, and providing feedback on the search strategy. The following are examples of this contribution:

“The assistance by Mrs. Morgan from the University of Cape Town library in formulating the search strategy is greatly appreciated.”

“The MEDLINE search strategy was developed by a librarian experienced in systematic review searching, and peer reviewed by another librarian using the PRESS standard.”

“The search strategy based on the combination of relevant terms was designed by a librarian.”

#### 2. Conducting the literature search (146 publications)

The second most common role played by LIS professionals, including those from South Africa (n=15), Ethiopia (n=2), Uganda (n=3), and Kenya (n=2), was conducting the literature search. LIS professionals provided guidance or assistance with conducting the search or performed the search themselves.

“The authors would like to thank Leila Ledbetter, biomedical librarian, for her assistance conducting the literature search. “ “Information specialists at the University of Cape Town Medical Library assisted with the literature search process.”

“The authors would like to thank Dilshaad Brey and Tamzyn Suliaman from the Health Sciences Library at the University of Cape Town for their time and support in developing and conducting the literature searches.”

“An expert librarian performed a search of PubMed/MEDLINE, Excerpta Medica Database, African Journals Online and African Index Medicus without any language restriction.”

#### 3. Full-text article retrieval (56 publications)

LIS professionals, including those from South Africa (n=9), Nigeria (n=1), and Ethiopia (n=3), provided consultation and advice for accessing and retrieving the full text of articles or obtained the full-text articles themselves.

“We thank Mr Devind Peter (Health Sciences Library, University of the Witwatersrand, Johannesburg, South Africa) for his kind assistance with the literature retrieval process.”

“We thank the Health Sciences Library at the University of Cape Town for assistance in obtaining full-text articles.”

“We gratefully acknowledge the Library staff of Usmanu Danfodiyo University, Sokoto for their advice on retrieval of full-text publications.”

#### 4. Electronic database access (33 publications)

LIS professionals, including those from South Africa (n=6), Tanzania (n=1), and Ethiopia (n=7), provided expertise with electronic database access.

“We acknowledge Tamzyn Suliaman, University of Cape Town's Health Sciences Librarian, for her tutorials and guidance on navigation of the different databases that were accessed for this review.”

“The authors thank the University of Dar es salaam (UDSM) Library by providing access to some bibliographical databases”

#### 5. Technical support (11 publications)

Some LIS professionals, including those from South Africa (n=9), provided technical support for the systematic review or meta-analysis process.

“The authors acknowledge Ms. Tamzyn Suliaman, UCT Libraries, Health Sciences, and Information Services Librarian, who provided technical support.”

“The primary investigator is indebted to Mrs. Morgan from the UCT library for the technical support afforded up to this time.”

#### 6. Database selection (8 publications)

LIS professionals also helped select electronic databases for the retrieval of relevant articles, but none were from Sub-Saharan African countries.

“An expert librarian at the paramedical library of the University of Montreal provided assistance for the selection of relevant databases.”

“We searched EMCARE instead of The British Nursing Index listed in our published protocol because our medical librarian advised us that the EMCARE database would contain more relevant information.”

#### 7. Article screening/selection (6 publications)

Some LIS professionals, including those from Ethiopia (n=1) and South Africa (n=1), helped screen and select articles for inclusion in systematic reviews and meta-analyses.

“The initial screening of references retrieved in the search was performed by two independent medical librarians to identify potentially relevant studies based on the titles and abstracts. “

“Alison A Kinengyere - conducted part of the literature search, screened articles, and extracted the data.”

#### 8. Reference management (4 publications)

Some LIS professionals, including those from South Africa (n=3), performed reference management in support of systematic reviews and meta-analyses.

“We thank Tamzyn Suliaman, University of Cape Town, South Africa, for technical support and assistance in the planning of the search strategy and management of references.”

#### 9. Reviewing protocol/manuscript draft/abstract (3 publications)

Some LIS professionals reviewed written components of systematic reviews and meta-analyses, including the protocol, manuscript draft, or abstract, but none were from Sub-Saharan African countries.

“The authors wish to thank Sarah Safrenek at the University of Washington Health Sciences Library for reviewing this protocol and assisting with the search strategy.”

#### 10. Teaching and training (2 publications)

Some LIS professionals, including one from South Africa, contributed to systematic reviews and meta-analyses by serving as teacher or trainer.

“We acknowledge Tamzyn Suliaman, University of Cape Town's Health Sciences Librarian, for her tutorials and guidance on navigation of the different databases that were accessed for this review.”

#### 11. Review design/conceptualization (2 publications)

Some LIS professionals, including one from South Africa (n=1), contributed to systematic review design or conceptualization.

“The authors thank Carol Mitta research reference librarian at Harvard Medical Library for her contribution to the study design and search strategy.”

#### 12. Data extraction (2 publications)

Some LIS professionals, including those from Uganda (n=1) and South Africa (n=1), performed data extraction for the systematic reviews or meta-analyses.

“Designed the search strategy, and contributed to abstract reviews and data extraction plans”

#### 13. Manuscript writing (1 publication)

One LIS professional, who was not from a Sub-Saharan African country, supported the systematic review process through manuscript writing.

“I. M. N. contributed to the article draft”

#### Relationships among collaboration type, first author's country affiliation, and LIS professionals' country affiliation

Most LIS professionals from Sub-Saharan Africa mentioned in systematic reviews and meta-analyses were associated with publications that had first authors from Sub-Saharan Africa (90.20%) and involved only Sub-Saharan African authors (61.66%) (no collaboration (0.98%), institutional collaboration (23.53%), national collaboration (18.63%), and intra-Sub-Saharan Africa collaboration (18.63%) (Table 2). On the other hand, most LIS professionals (97.91%) mentioned in publications involving international collaboration were from outside Sub-Saharan Africa.

**Table 2:** Collaboration type, first author's country, and LIS professionals' country affiliations

		LIS professionals' country affiliations			Total
		Within Sub-Saharan Africa (n=102)	Outside Sub-Saharan Africa (n=191)	Hybrid (n=2)	
First author's address	Within Sub-Saharan Africa	92 (90.20%)	26 (13.61%)	1 (50%)	119
	Outside Sub-Saharan Africa	4 (3.92%)	103 (53.93%)	0	107
	Hybrid	6 (5.88%)	62 (32.46%)	1 (50%)	69
Collaboration type	No collaboration	1 (0.98%)	2 (1.05%)	0	3
	Institutional collaboration	24 (23.53%)	1 (0.52%)	0	25
	National collaboration	19 (18.63%)	1(0.52%)	0	20
	Intra-Sub-Saharan Africa collaboration	19(18.63%)	0	1 (50%)	20
	International collaboration	39 (38.34%)	187 (97.91%)	1 (50%)	227
	Total	102	191	2	295

Spearman's rho correlation analysis was performed to determine the relationships between LIS professionals' country affiliation and two other variables: the first author's country affiliation and collaboration type. There was a moderate positive relationship between LIS professionals' country affiliation and the first author's country affiliation (r_s_=0.648, p<0.05). Likewise, there was a moderate positive relationship between LIS professionals' country affiliation and collaboration type (r_s_=0.643, p<0.05). These results indicate that LIS professionals from Sub-Saharan Africa were more likely to contribute to systematic reviews and meta-analyses that had first authors from a Sub-Saharan African country and involved only authors from the region.

## DISCUSSION

The systematic reviews and meta-analyses analyzed in this study were authored by at least one researcher affiliated with an institution in Sub-Saharan Africa. We found that LIS professionals from institutions outside Sub-Saharan Africa contributed to systematic reviews and meta-analyses twice as often as LIS professionals from institutions within the region. Hence, there appears to be a need for further research to investigate why LIS professionals in the Sub-Saharan Africa region contribute less to evidence-based biomedical research in their own region. Perhaps there are training gaps for medical and health information professionals in Sub-Saharan Africa as noted in earlier studies [17,18], which could be filled by including evidence-based research courses in the curriculum for information schools in the region.

We identified thirteen distinct roles of LIS professionals in systematic reviews and meta-analyses, supporting earlier studies. Although Beverley, Booth, and Bath noted that the roles of LIS professionals in systematic reviews have evolved from simply acting as “evidence locators” and “resource providers” [8], we found that the most common role attributed to LIS professionals was helping with the search strategy. This search-related role is expected of LIS professionals and was also the most frequently occurring role noted in previous studies [4,7,8]. Furthermore, consistent with McGowan and Sampson as well as Harris, who discussed how expert searchers are an essential part of the systematic review team [2,6], searching was key among the roles described in our study. However, consistent with Spencer and Eldredge [4], we found that LIS professionals also contribute to systematic reviews and meta-analyses through other roles such as teaching and training, designing and conceptualizing reviews, and reviewing protocols.

Our analysis shows LIS professionals from Sub-Saharan African countries contributed in the thirteen classes of contributions that were identified. Noteworthy is the frequent contributions made by LIS professionals from South Africa. For instance, LIS professionals from South Africa contributed in all ten roles in which Sub-Saharan African LIS professionals were mentioned. Also, South African researchers authored almost 50% of all systematic reviews and meta-analyses included in this study, and LIS professionals from South Africa were mentioned four times more than those from any other Sub-Saharan African country and two times more than all other Sub-Saharan African countries together. In practice, this implies that South Africa's position of leadership in Sub-Saharan Africa requires that it provides help to other Sub-Saharan African countries. For instance, the University of Pretoria in South Africa hosted an all-expenses-paid residential continuous development program for librarians in five Sub-Saharan African countries (South Africa, Nigeria, Uganda, Ghana, and Tanzania), through which 192 participants were trained between 2013 and 2016 [19].

Considering the regional output of systematic reviews and meta-analyses, two major patterns emerged. First, the contribution of South Africa was markedly higher than that of other countries in Sub-Saharan Africa. This is not surprising given that South Africa is often ranked as the highest performer in research in Africa [20,21]. Going forward, Sub-Saharan Africa needs more assistance and leadership from South Africa (because of their leadership position in evidence-based biomedical research as shown in this study) alongside other countries such as the US, the UK, Canada, the Netherlands, and Australia that contributed significantly to sampled systematic reviews and meta-analysis. Second, LIS professionals from some highly ranked Sub-Saharan African countries like Cameroon, Ghana, Zimbabwe, Malawi, and Zambia were not noted to make any contribution to systematic reviews and meta-analyses. This suggests that approaches to improving African LIS professionals' participation in evidence-based research cannot be generalized across all countries. While LIS professionals in some countries have not started to participate in evidence-based research, others have started but need additional professional development.

Furthermore, we found that LIS professionals from Sub-Saharan Africa were more likely to contribute to systematic reviews and meta-analyses with a Sub-Saharan African first author and for which all authors were from Sub-Saharan Africa. This finding may be related to that of Asubiaro, who found that LIS publications with African first authors received fewer citations than publications with first authors from other regions of the world, whereas LIS publications involving international collaboration between Africa and other regions of the world received more citations [21]. This suggests that LIS professionals from Sub-Saharan Africa were less likely to be consulted in international collaboration research and studies with foreign first authors, although research shows that these types of studies from Sub-Saharan Africa are more cited than those involving internal collaboration with first authors from the region [21]. On the other hand, researchers preferred to invite LIS professionals from outside Sub-Saharan African countries when there is collaboration between researchers within and outside Sub-Saharan African countries.

While we advocate for more training for LIS professionals from Sub-Saharan Africa, it is also important to point out that there is a need to characterize the content of LIS curricula from this region in future studies. However, anecdotal information indicates that there are very few continuous professional development programs in Sub-Saharan Africa for working LIS professionals. Traveling to North American and European countries is not affordable for most LIS professionals in the region because of the near absence of funds for training and conference attendance. In light of this, we recommend that national library associations, including the Association for Health Information and Libraries in Africa and the African Library and Information Associations and Institutions, collaborate with LIS associations outside the region, such as the Medical Library Association, International Federation of Library Associations and Institutions, European Association for Health Information and Libraries, and International Congress of Medical Librarianship, for training, retraining, advocacy, mentoring, and collaboration. This collaboration could include hosting trainers from other parts of the world in Sub-Saharan African countries instead of the current model of bringing LIS professionals from Sub-Saharan Africa to the Western countries on scholarship or fellowship. This way, more LIS professionals can be trained in a more cost-effective manner. Also, trainers can directly experience some of the challenges that LIS professionals face while teaching. This study shows the strong influence of LIS professionals from the US, Canada, the Netherlands, the UK, and Australia in evidence-based research from the Sub-Saharan Africa region. Thus, these countries arguably house the most influential institutions that could be included in these potential training collaborations.

Because only author affiliations, methodology, and acknowledgments sections were searched for information about the contribution of LIS professionals, it is possible that LIS professionals' contributions were underreported. Therefore, we recommend that authors give more recognition to the roles that LIS professionals play in evidence-based research. Also, manual content analysis of a large number of full-text articles could have resulted in some omissions.

## Data Availability

All datasets associated with our results are publicly available in Mendeley Data at http://dx.doi.org/10.17632/xkry6rjtjg.3.

## SUPPLEMENTAL FILES

Search strategy executed on PubMed/MEDLINE
