# Supplementary material for: Evidence-based biomedical research in Sub-Saharan Africa: how library and information science professionals contribute to systematic reviews and meta-analyses
Source: J Med Libr Assoc. 2022 Jan 1;110(1):72–80. doi: 10.5195/jmla.2022.1249 (PMC8830396; doi:10.5195/jmla.2022.1249)
Supplement: Supplementary file 1 — Search strategy executed on PubMed/MEDLINE [file jmla-110-1-72-s01.docx]

**Search strategy executed on PubMed/MEDLINE**

| **Step #1:** | *Nigeria[Affiliation] OR South Africa[Affiliation] OR Ghana[Affiliation] OR Tanzania[Affiliation] OR Kenya[Affiliation] OR Rwanda[Affiliation] OR Botswana[Affiliation] OR Cameroun[Affiliation] OR Senegal[Affiliation] OR Angola[Affiliation] OR Uganda[Affiliation] OR Mali[Affiliation] OR Sierra Leone[Affiliation] OR Ivory Coast[Affiliation] OR Ethiopia[Affiliation] OR Lesotho[Affiliation] OR Zambia[Affiliation] OR Zimbabwe[Affiliation] OR Namibia[Affiliation] OR Guinea[Affiliation] OR Mauritius[Affiliation] OR Mozambique[Affiliation] OR Niger[Affiliation] OR Seychelles[Affiliation] OR Burkina Faso[Affiliation] OR Burundi[Affiliation] OR Cape Verde[Affiliation] OR Cameroon[Affiliation] OR Central African Republic[Affiliation] OR Chad[Affiliation] OR Comoros[Affiliation] OR Democratic Republic of Congo[Affiliation] OR DR Congo[Affiliation] OR Djibouti[Affiliation] OR Cote D'ivoire[Affiliation] OR Congo[Affiliation] OR Equatorial Guinea[Affiliation] OR Eritrea[Affiliation] OR Gabon[Affiliation] OR Guinea-Bissau[Affiliation] OR Madagascar[Affiliation] OR Congo Republic[Affiliation] OR Sao Tome and Principe[Affiliation] OR Swaziland[Affiliation] OR Togo[Affiliation] OR Benin[Affiliation] OR Liberia[Affiliation] OR Namibia[Affiliation] OR Gambia[Affiliation] OR (Cent Afr Republ[Affiliation]) OR (Equat Guinea[Affiliation]) OR (Papua N Guinea[Affiliation]) OR (Sao Tome E Prin[Affiliation]) OR Principe[Affiliation] OR Sao Tome E Principe[Affiliation]* |
| --- | --- |
| **Step #2:** | Set Filter to*: Meta-Analysis[ptyp] OR systematic[sb]* |
| **Step #3:** | Text word search *systematic review[Text Word] OR meta-analysis[Text Word] OR meta analysis[Text Word]* |
| **Step #4:** | Set publication date to: *"2014/01/01"[PDAT] : "2019/12/31"[PDAT]* |
| **Query:** | (Step #1) AND (Step #2 OR Step #3) AND (Step #4) |
